# Mediation of Problematic Use in the Relationship Between Types of Internet Use and Subjective Well-Being in Schoolchildren

**DOI:** 10.3389/fpsyg.2021.641178

**Published:** 2021-03-16

**Authors:** Gonzalo Donoso, Ferran Casas, Andrés Rubio, Cristian Céspedes

**Affiliations:** ^1^Research Institute on Quality of Life, Universitat de Girona, Girona, Spain; ^2^Innovation Center, Pontificia Universidad Católica de Chile, Santiago, Chile; ^3^Programa de Doctorado en Educación y Sociedad, Universidad Andres Bello, Santiago, Chile; ^4^Facultad de Economía y Negocios, Universidad Andres Bello, Santiago, Chile; ^5^Facultad de Psicología, Universidad Diego Portales, Santiago, Chile; ^6^Facultad de Administración y Economía, Universidad de Santiago de Chile, Santiago, Chile

**Keywords:** subjective well-being, belonging, personal well-being index, problematic Internet use, digital divide

## Abstract

Subjective well-being is a broad category of phenomena that includes people’s emotional responses, domain satisfactions, and global judgments of life satisfaction. This research investigates how schoolchildren’s subjective well-being is affected by the different types of technology use, in personal contexts, and, concurrently, whether these effects are different when the use of technology is problematic. The central hypotheses are as follows: (1) the use of the Internet affects the subjective well-being of schoolchildren negatively only when this use is problematic and (2) the effect on subjective well-being is different according to the type of Internet use. To respond to the objectives of the research, a survey was applied to 15-year-old adolescents (2,579 cases), distributed in 330 public schools, beneficiaries of a government program for the delivery of personal computers and Internet for a year. The different uses of the Internet were measured using frequency scales by type of activity (social, recreational, and educational). Problematic use scale measured the perception of negative consequences of the intensity of Internet use on a daily basis. Subjective well-being was measured by the Personal Well-Being Index-School Children (PWI-SC). Subsequently, for analytical purposes, three simple mediation models were created, whose dependent variable was PWI-SC, while its independent variables were Internet use scales differentiated by purpose (social, recreational, and educational) and problematic use as a mediating variable, as well as attributes of the subjects and their social environment, which were incorporated as control variables. The main results show that only if Internet use is expressed as problematic does it negatively affect subjective well-being. On the contrary, when the use of the Internet is not problematic, the effect is positive and even greater than the simple effect (without mediation) between these two variables. This finding is relevant, since it allows us to provide evidence that suggests that, when studying the effect that the intensity of the Internet, firstly, one must consider the mediating effect exerted by the network’s problematic use and, secondly, that not all types of use have the same impact. Therefore, it is useful to enrich the discussion on subjective well-being and social integration of schoolchildren in the digital age.

## Introduction

Over the last decades, the relentless proliferation of digital technologies has profoundly transformed and impacted all human activities. We understand as digital development the degree to which these technologies penetrate society, considering the scope and depth of their deployment, and whether this is reflected in the increase in the possibilities that the population has to accessing, using, and learning about them (Minges, 2005; ITU, 2017; van Deursen et al., 2017). In this regard, experts on the field seem to agree that access to technology is not equitably distributed between countries, territories, or among the different members of society. This initial barrier, which hinders equitable access to technology, is called the first-generation digital divide (Selwyn, 2004, 2010; Talaee and Noroozi, 2019; Van Dijk, 2019). More recently, the specialized discussion has broadened the scope of the definition of the first-generation digital divide, since, in order to conceptualize the scope of digital development and its relationship with social integration, it is necessary to pay attention not only to the possibilities of access but also to the capacities that people should have to take advantage of them. This subsequent stage is called the second-generation digital divide (Büchi et al., 2016; Scheerder et al., 2017). Although it is evident that there is more and more access to mobile devices, computers, and Internet connectivity, as a result of the technological development, the existence of a first- and second-generation digital divide is a global ongoing problem, especially in developing countries. For this reason, countries have found in the school system an effective way to reduce it through various programs of access to technology at schools or through the delivery of digital equipment to students (Cabello and Claro, 2017; Cabello et al., 2020). In the case of Chile, the 1:1 modality was not massively adopted in its period of greatest popularity, in the mid-2000s, since the country opted to maintain technology for educational purposes in the institutional context of the school. Later, in 2015, this policy became universal for the case of seventh-grade students in public schools (Severin, 2016; Claro and Jara, 2020).

This significant expansion of digital technology access and the consequent increase in the intensity of its use have generated growing concern about the possible effects that it could have not only on the academic performance of adolescents, whether positively (Kim et al., 2017) or negatively (Bulut and Cutumisu, 2018), but also in other areas considered increasingly relevant, such as their subjective well-being (López et al., 2014, 2017; Alfaro et al., 2016; OECD, 2017, 2019). Subjective well-being research is one of the most prolific fields in the scientific literature in human and social sciences in recent years (Diener et al., 2018).

Theoretically speaking, subjective well-being is made up of cognitive judgments and emotional responses (Diener et al., 2018), and it is defined as the different assessments that people make about their life, the events that happen to them, their bodies and minds, and the circumstances in which they live (Diener, 2006). It is a broad category of phenomena that includes people’s emotional responses, domain satisfactions, and global judgments of life satisfaction (Diener et al., 1999).

In the case of school-age children and adolescents, it is essential to investigate what factors may affect this construct, since the experiences lived in this period are not only very relevant in their present lives but also determining factors for the development of their cognitive, social, and emotional skills (Ning et al., 2013) and are key to the formation of mentally healthy adults. They also play a role in the level of satisfaction these people will have toward life (Casas et al., 2020), their future fulfillment projects, relational and self-perception frameworks (Cobo-Rendón et al., 2020), and their sense of belonging (Berryman and Eley, 2019), among other fundamental elements for life in society (Bilbao et al., 2014).

In this regard, the existing literature on the possible effects between access and use of technology and subjective well-being in children and adolescents shows ambivalent results (Ivie et al., 2020; Kardefelt-Winther et al., 2020; Young et al., 2020). Primarily, it is possible to identify evidence that indicates that the access and use of digital technologies benefit the social integration of this segment of the population, their sense of belonging, as well as their level of connection and understanding between members of their social environment (Walsh et al., 2020). At the same time, it allows children and adolescents a better use of their time, both productive and free; it increases opportunities for learning and personal development (Hollingworth et al., 2011; Hatlevik and Christophersen, 2013; van Deursen and van Dijk, 2014); and it improves their mental well-being (Stephens-Reicher et al., 2011; Clifton et al., 2013). In this same trend, greater access to information and communications technology (ICT) may lead to acquiring technical skills and increasing self-esteem and social capital (Best et al., 2014). Also, evidence claims that students’ subjective well-being is positively linked to their school’s digital development and their social well-being and the school climate they are in (Donoso et al., 2021). Furthermore, a higher frequency of Internet use, given that context variables are adequately controlled, is not associated with lower subjective well-being (Paez et al., 2020).

In contrast, it is also possible to find negative associations between the use of digital technologies and personal well-being or mixed associations (Odgers, 2016; Przybylski and Weinstein, 2017; Orben and Przybylski, 2019; Young et al., 2020). Thus, there is literature that indicates that the use of the Internet by schoolchildren is often associated with risks related to loneliness (Song et al., 2014; Ho et al., 2017), social anxiety (Buyukbayraktar, 2020; Traş and Gökçen, 2020), cyberbullying (Devine and Lloyd, 2012; Casas et al., 2013; Viner et al., 2019; Craig et al., 2020; Garaigordobil et al., 2020), unsafe sexual behaviors (McBride, 2011; Vannucci et al., 2020), and psychological pathologies (Borzekowski, 2006; McBride, 2011). Other works suggest that a high use of social networks and online games are related to low subjective well-being (Devine and Lloyd, 2012) or addictive behaviors (García-Oliva et al., 2017; Marengo et al., 2020). It should be noted that it is possible to identify a concordance within the evidence that shows a negative association between the use of technologies and subjective well-being, which occurs more strongly in girls than in boys when observed (Twenge and Martin, 2020).

Added to this open discussion is the fact that, in order to advance in the understanding of the phenomenon, it is essential to analyze the different social contexts where adolescents develop, considering a broad perspective to understand better how this relates to the use of digital technologies, subjective well-being, belonging, and social integration (Helsper et al., 2015; Helsper, 2017; Helsper and van Deursen, 2017; Livingstone et al., 2017; Boer et al., 2020). For this reason, recent research in the field no longer focuses solely on what happens inside schools but in all social spaces in which children and adolescents interact (Craig et al., 2020), both concrete and virtual. Particular emphasis is placed on screen time and Internet use, particularly on social networks (Büchi et al., 2019; George et al., 2020), at different times of the day, both school and personal time (Beyens et al., 2020). This use, when it becomes too intensive and frequent, can be considered excessive or problematic.

However, the complexity of the phenomenon makes its proper measurement difficult, despite the fact that various conceptualizations classify the problematic use of technologies in the field of addictions (Ho et al., 2017), whether behavioral, to the Internet, mobile phone, or social networks (Martín-Perpiñá et al., 2019), and that various proposals have come up on how to define problematic appropriately (García-Oliva et al., 2017; Büchi et al., 2019) or excessive use (Malo-Cerrato et al., 2018). In this sense, the literature indicates that in order to classify the use of technology as problematic, not only the intensity and frequency of such use should be considered but also how it affects the normal development of people’s daily lives, especially in personal and social aspects (Castellana Rosell et al., 2007; Viñas Poch, 2009; Smahel et al., 2012; Rial and Gómez Salgado, 2018).

Since the concept of problematic use of technology is still inexact, it is not clear whether the type or purpose of its use has any differentiated effect on the subjective well-being of adolescents or whether this would also have a negative, harmless, or even positive effect when it is intense but still does not qualify as problematic.

Therefore, the research questions that lead this work are essentially the following: (1) Does the use of the Internet, in personal contexts, always affect schoolchildren’s subjective well-being negatively or only when it is problematic? (2) Do the effects on subjective well-being differ when different types of Internet use are examined separately? The working hypotheses are the following: (1) the use of the Internet negatively affects the subjective well-being of schoolchildren only if the use falls under the category of problematic and (2) the effect on subjective well-being is different according to the type of use from Internet. Although these hypotheses are plausible according to the literature, there is still not enough evidence to support them since previous research has not delved further into whether there are differentiated effects by type of use.

The objective of this study is to provide evidence on how the access and use of digital technologies, distinguishing between their purposes and problematic nature, are related to the subjective well-being of adolescents.

## Materials and Methods

The research design is framed within a quantitative methodological approach, and it links the concepts of access and use of digital technologies (personal computer and Internet) and subjective well-being, distinguishing them by their purpose and problematic nature.

As a source of information, the data obtained by the Ministry of Education of Chile (MoE) were used in the framework of the evaluation of the implementation of a government program, which consists of the provision of personal computers and Internet access, through mobile broadband for 12 months, with an average speed of 700 Kbps download and 256 Kbps upload (DIPRES, 2018). It should be noted that the equipment delivered becomes the property of the beneficiary, and they do not have to return it. This public policy, currently called “Me Conecto para Aprender” or MCPA (In English, “I get connected to Learn”), was started in 2008 and later expanded in 2015, and it currently focuses on two large groups of the school population. On the one hand, seventh-grade primary school students who attend private state-subsidized schools show an outstanding academic record and belong to the poorest sectors of the population. And, on the other hand, seventh grade public school students, universally, that is, regardless of their socioeconomic status or their academic performance.

### Sample

The universe was made up of student beneficiaries of the program described in the years 2015 and 2016. These students received the equipment and the respective Internet connection. The effective sample size reached 2,579 cases, of which 44.4% corresponded to female students and 55.6% to male, and whose average age was 14.23 years (SD = 0.97). Regarding the distribution by cohort of admission to the program, 45.5% of the cases correspond to students who attended the seventh grade of primary education during 2015 and 54.5% in 2016. Out of the total sample, 79.4% kept the computer delivered and 85.0% had an Internet connection either through the device delivered by the program or another available at home. Likewise, 8.5% did not have access to any computer at home, as the equipment delivered no longer worked, had been lost, sold, or stolen, and there was no other equipment available either for personal or family use.

The cases belong to 330 school establishments (87.9% urban and 12.1% rural) that receive public funding [86.7% public and 13.3% government-dependent private (voucher)], located in the regions with the highest concentration of population throughout the three geographical macro-areas of the country (12.8% north, 70.5% center, and 16.7% south). A probabilistic, two-stage, and stratified sample was used, where the first-level units were the establishments and the final units were the students, with national representation, and with an estimation error corresponding to ±1.91% with a 95% confidence level and maximum variance. The sampling frame was built using the national school enrollment registry of the Ministry of Education of Chile (2017) and was complemented with data from the National School Vulnerability Index (IVE) to include the average socioeconomic status (SES) of the school attended by the subjects that constituted the units of analysis (López et al., 2017). The IVE, whose score ranges from 0 to 100%, where a higher score indicates greater vulnerability, is constructed from social, economic, health, and academic variables of the students and their homes (Ñanculeo and Merino, 2016). Said variables account for the situation of social or educational risk that they must face. Given the deep segregation of the Chilean school system, the consequent socioeconomic homogeneity of students within each school can be used as an indicator that mirrors the reality of each student who attends it (González and Fernández-Vergara, 2019; Bellei et al., 2020).

Given that the cases come from the beneficiaries of a focused public policy, the sample tends to prevail with students belonging to the lowest SESs. Indeed, 56.4% of the sample cases attend schools with high social vulnerability and 43.6% attend schools with low vulnerability (using the IVE score = 80% as a cutoff criterion), the mean of the sample being 70.51%.

### Instrument

The instrument used was previously validated by experts from the MoE and academic institutions. In addition, the results obtained in a pilot application to a sample of 38 cases, belonging to three schools, carried out in June 2017, were considered. The pilot cases’ selection criteria were the same as those used in the massive sample, and they were not part of the final database. This phase allowed observing the behavior of the questionnaire in aspects related to its applicability, duration, comprehension, and non-response per item rate. Thanks to this, specific adjustments, validated by experts, were made to improve the final instrument before being applied on a large scale.

The instrument considered dimensions related to the program’s implementation, considering selection, delivery, and operating status of the equipment, as well as support mechanisms, habits of use, parental mediation, problematic use, and subjective well-being.

The Personal Well-Being Index-School Children (PWI-SC), designed by Cummins et al. (2003), was used to measure the subjective well-being of the students. Specifically, the Spanish version of the PWI-SC7 was used, adapted and validated by Bilbao et al. (2014), which investigates the level of satisfaction that adolescents have regarding areas related to their health, standard of living, achievements, interpersonal relationships, sense of agency, and satisfaction with themselves and with future security. The response range extends from “Totally Dissatisfied” (score = 0 points) to “Totally Satisfied” (score = 10 points). In the present study, its application registered a high internal consistency (*α* = 0.91) and a configuration of a single factor (KMO = 0.91; Bartlett = 10,016.456, *p* < 0.001). Said results prove consistent with those obtained in previous applications of the scale at the national level (Casas and Bello, 2012; Bilbao et al., 2014). For the calculation of the described scale, the raw scores of its items were added, and then the simple average was obtained.

To measure the use of technology, specifically, Internet use, three scales were applied, designed to record activities with specific purposes: social, recreational, and educational. The questions of the three scales were formulated regarding the frequency of use in the area consulted over the previous month, with a response range extending from “Never” (score = 1) to “Every day, several times a day” (score = 6). To calculate the total score of each scale, the raw scores of its items were added, and then the simple average was obtained. To analyze its factorial structure, the Principal Components Method with Varimax rotation was used to obtain the simplest and most coherent structure possible.

The scale of social use of the Internet (SUIS) is made up of six items. It considers activities related to participation in social networks and the use of instant messaging applications and video calls to communicate with peers or relatives, posting or sharing photos, videos, music, or personal interests. Its application registered a high internal consistency (*α* = 0.81) and a configuration of a single factor (KMO = 0.80; Bartlett = 5,248.359, *p* < 0.001), which explains 52.28% of the total variance of the scale.

The Recreational Internet Use Scale (RUIS) considers four items and groups together the reproduction and consumption of content, both written and multimedia, including music and audiovisual material, as well as playing online video games. Its application registered an acceptable internal consistency (*α* = 0.69) and a configuration of a single factor (KMO = 0.67; Bartlett = 2,351.803, *p* < 0.001), which explains 54.25% of the total variance of the instrument.

The items of both scales were originally developed by the Global Kids Online project (Livingstone and Haddon, 2009) and adapted to the Spanish language by Cabello and Claro (Cabello et al., 2020) for their application in the local context.

The educational use of the Internet scale (EUIS) was made up of 12 items, including school practices and informal learning. School practices consider homework, such as making presentations, assignments, and research, either individually or in groups, and communicating with other students and teachers for educational purposes (MINEDUC, 2013; Jara et al., 2015; Fraillon et al., 2020). In the case of informal learning activities (Helsper et al., 2015), they include the use of videos or tutorials to acquire skills of personal interest and the use of resources available on the Internet to learn or delve into unsolicited subjects in the school context. Its application registered high reliability (*α* = 0.88) and a configuration that groups the items into three factors (KMO = 0.88; Bartlett = 3559.650, *p* < 0.001), according to what was expected, and that, combined, explain the 66.40% of the total variance of the instrument (homework, 26.35%; communication, 21.24%; and informal learning, 18.81%).

Finally, to measure problematic Internet use (PUIS), the scale developed by Smahel et al. (2012), which investigates the perception of negative consequences of the intensity of Internet use on a daily basis, was employed. Through five items, it considers aspects related to alternations in sleep and eating, conflicts with peers and family, low school performance, inability to self-regulate, and awareness of excessive use. The questions were formulated in reference to the last year, and they regarded the frequency of occurrence of episodes of problematic use, with the responses ranging from “Never” (score = 1) to “Always” (score = 5). Its application registered high internal consistency (*α* = 0.81) and a configuration of a single factor (KMO = 0.81; Bartlett = 4,020.315, *p* < 0.001) that explains 57.52% of the total variance of the instrument. Like the previous scales, this one was also adapted and translated into Spanish to ensure an adequate application to the study population.

### Procedure

The production of the information was carried out in person at the schools that made up the sample, during regular class hours, after contacting the corresponding managers and under the supervision of a facilitator. All necessary procedures were followed to obtain the administrative authorization of the schools, informed consent, safeguarding of confidentiality, and voluntary participation, according to the ethical standards required by the MoE and the university in charge of collecting the information. The application period was between October and December 2017.

Access to the corresponding data sources was obtained through a formal request to the MoE, the entity responsible for the study. The information was processed using IBM SPSS Statistics 24 and the PROCESS Macro for SPSS v2.10 modeling tool. The information collected from IVE was added to the database, using the Role Database (RBD) as a key field, which operates as the sole identifier of each school in the country.

### Analysis

Once the database was consolidated and refined, descriptive analyses of the main study variables were carried out. After that, tests of differences in means between sociodemographic attributes, the PWI (PWI-SC), and PUIS were performed. For this purpose, the PUIS was recoded using the average score observed in the sample as the grouping criterion. The resulting auxiliary variable was called level of problematic Internet use, and it has two categories, low and high, depending on the location of each subject on said scale (above or below the grand mean). Pearson and Spearman correlations were also calculated depending on the measurement level of the involved variables.

Subsequently, to further contrast the study’s hypotheses, three multivariate models of simple mediation were constructed (Hayes, 2018), which assumed the subjective well-being of the students (PWI-SC7) as a dependent variable and PUIS as a mediating variable. Each model is distinguished by incorporating a specific use scale to the set of independent variables, with the purpose of separately analyzing the effect of the type of technology use on subjective well-being. Thus, the first model incorporated the social use scale (SUIS) into the group of explanatory variables; the second, the recreational use scale (RUIS); and the third, the educational use scale (EUIS). Additionally, individual control variables of the subjects (sex, age, and cohort) were incorporated into each model and, to control the group effect, the SES of the school was included. The detail of the variables considered in the analyses can be seen in Table 1. In the case of categorical variables, fictitious variables (dummies) were constructed so that they could be included in the respective models. Both the independent and control variables were selected based on their theoretical and empirical importance for this investigation. A BCa bootstrapped CI based on 5,000 samples was used to calculate the confidence intervals of all the models used.

**Table 1:** Contextual and control variable description.

Level	Variable	Level of measurement	Categories
School	Socioeconomic status (SES): retrieved from National School Vulnerability Index (IVE-SINAE)	Nominal	0 = High
			1 = Low
Individual (student)	Sex	Nominal	0 = Men
			1 = Women
	Age	Scale	
	Cohort: year of entry to the program and time since the student received the computer	Nominal	0 = 2015
			1 = 2016

## Results

The results of the descriptive analysis (Table 2) indicate that the subjects that made up the sample registered an average PWI of 8.04 points (SD = 1.84), being 8.17 higher in men (SD = 1.76) than their female peers, 7.88 (SD = 0.97). This difference is statistically significant [*t*(2509) = 3.953, *p* < 0.001, D = −0.16].

**Table 2:** Descriptive statistics.

	N	Minimum	Maximum	Mean	SD
Personal Well-Being Index (PWI)	2,511	0.00	10.00	8.04	1.84
Social use of Internet (SUIS)	2,511	1.00	6.00	3.31	1.26
Recreational use of Internet (RUIS)	2,511	1.00	6.00	3.86	1.19
Educational use of Internet (EUIS)	2,511	1.00	6.00	3.07	0.96
Problematic use of Internet (PUIS)	2,511	1.00	5.00	1.88	0.83
Age	2,511	12	18	14.24	0.97

SD, standard deviation.

In the case of the Internet-social use (SUIS), recreational (RUIS), and educational (EUIS) scales, it is observed that the mean is 3.31 (SD = 1.26), 3.86 (SD = 1.19), and 3.07 (SD = 0.96), respectively. Additionally, the mean of the problematic Internet-use scale is 1.88 (SD = 0.83).

When comparing the differences in the scores obtained by male students and female students in the four scales, it is found that the means are very similar in the SUIS (male = 3.31, SD = 1.25; female = 3.31, SD = 1.27), EUIS (male = 3.05, SD = 0.97; female = 3.09, SD = 0.95), and PUIS (male = 1.90, SD = 0.82; female = 1.84, SD = 0.84) and slightly higher in the case of male students on the RUIS scale (male = 3.92, SD = 1.19; female = 3.78, SD = 1.19). Finally, when applying tests of differences of means, using Student’s *t*(*α* = 0.05), these were not statistically significant for SUIS, *t*(2509) = 0.075, *p* = 0.940; EUIS *t*(2509) = 1.124, *p* = 0.261; and PUIS, *t*(2509) = −1.683, *p* = 0.092; but it was in the case of RUIS, *t*(2509) = −2.881, *p* = 0.004, D = −0.14, although it is a minor effect.

When conducting mean difference tests (Table 3), using Student’s *t*(*α* = 0.01), comparing the levels of PUIS (categorized as high and low), it is possible to see that students who present low problematic use evidence higher subjective well-being, expressed in PWI score, regardless of the grouping variable through which the contrast is performed. That is, the boys and girls who register low problematic use show an average subjective well-being 6.8% higher than their peers with high problematic use of technology when comparing within each sex, SES group, or cohort of admission to the MCPA program. The differences are statistically significant (*p* < 0.001) in all the disaggregations examined.

**Table 3:** Mean difference in Personal Well-Being Index.

		Problematic use of ICT level	
		Low	High
Sex	Women	8.13	7.52^**^
	Men	8.41	7.87^**^
SES	High	8.30	7.77^**^
	Low	8.27	7.67^**^
Cohort	2015	8.10	7.74^**^
	2016	8.43	7.70^**^

ICT, information and communications technology; SES, socioeconomic status.

** Difference is significant at the 0.01 level (two-tailed).

In this sense, it is possible to corroborate the trend that female students tend to register lower subjective well-being than their male counterparts, which, in this case, is maintained regardless of whether they show low (male students = 8.41, female students = 8.13) or high problematic use of Internet (male students = 7.87, female students = 7.52). This relationship is also observed when comparing by SES, where those students with a high SES present higher subjective well-being than their peers in the lower group, regardless of whether they register a low PUIS (high SES = 8.30, low SES = 8.27) or high (high SES = 7.77, low SES = 7.67).

The results of the bivariate correlations between the scales (Table 4) indicate that subjective well-being (PWI) is significantly and positively related to the SUIS (*r* = 0.09), RUIS (*r* = 0.10), and EUIS (*r* = 0.15) and negatively with the PUIS scale (*r* = −0.15). All the correlations, either positive or negative, are significant at the 0.01 (two-tailed) level.

**Table 4:** Correlation matrix for the central variables of the study (*n* = 2,511).

S. No.		1. PWI	2. SUS	3. RUS	4. EUS	5. PUS
1.	Personal Well-Being Index (PWI)	1				
2.	Social use of Internet Scale (SUIS)	0.09^**^	1			
3.	Recreational use of Internet Scale (RUIS)	0.10^**^	0.63^**^	1		
4.	Educational use of Internet Scale (EUIS)	0.15^**^	0.53^**^	0.52^**^	1	
5.	Problematic use of Internet Scale (PUIS)	−0.15^**^	0.18^**^	0.16^**^	0.10^**^	1

** The correlation is significant at the 0.01 level (bilateral).

The correlations between the central variables of the study and the sociodemographic variables present different results (Table 5). In the case of the PWI, there is a positive and significant correlation between subjective well-being and income cohort (rho = 0.06), that is, in a shorter time elapsed since the student received the MCPA computer. The relationship is also positive with the group made up of male students (rho = 0.08). On the contrary, it is negatively correlated with the age of the subjects (*r* = −. 08). All the correlations, either positive or negative, are significant at the 0.01 (two-tailed) level.

**Table 5:** Correlation matrix for the central variables of the study and the sociodemographic variables (*n* = 2,511).

		Cohort	SES	Sex	Age
Personal Well-Being Index (PWI-SC7)	Correlation	0.06^**^	0.001	0.08^**^	−0.08^**^
	Sig. (two-tailed)	0.00	0.75	0.00	0.00
Social use of Internet Scale (SUIS)	Correlation	−0.02	0.06^**^	−0.01	0.02
	Sig. (two-tailed)	0.34	0.00	0.79	0.46
Recreational use of Internet Scale (RUIS)	Correlation	0.03	−0.05^**^	0.07^**^	−0.03
	Sig. (two-tailed)	0.14	0.02	0.00	0.11
Educational use of Internet Scale (EUIS)	Correlation	−0.01	−0.04^*^	−0.02	−0.00
	Sig. (two-tailed)	0.49	0.03	0.26	0.83
Problematic use of Internet Scale (PUIS)	Correlation	−0.00	−0.04	0.04^*^	−0.00
	Sig. (two-tailed)	0.83	0.07	0.03	0.91

All correlations correspond to Spearman’s coefficients (rho), except in the case of age variable, which corresponds to Pearson’s coefficient (*r*). SES, socioeconomic status.

** Correlation is significant at the 0.01 level (two-tailed).

* Correlation is significant at the 0.05 level (two-tailed).

Regarding Internet use, both the SUIS (rho = −0.06, *p* < 0.001), RUIS (rho = −0.05, *p* < 0.005), and EUIS (rho = −. 04, *p* < 0.005) are negatively correlated with SES. In other words, as the SES increases, the frequency of technology use by students tends to decrease. Likewise, both the RUIS (rho = 0.07, *p* < 0.001) and the PUIS (rho = 0.04, *p* < 0.005) are positively correlated with the group made up of male students. In other words, boys tend to use the Internet more intensively for recreational purposes and, at the same time, have more problematic use than girls. It should be noted that the cohort and SES variables are ordered from lowest to highest in accordance with the original grouping categories.

Afterward, the results of the three simple mediation models are presented, which, in turn, are made up of two sub-models. For each, there is a path diagram with the regression coefficients of each of the components of the different pathways presented by the model, considering the indirect effect, the direct effect, and the total effect over PWI.

**Figure 1:**
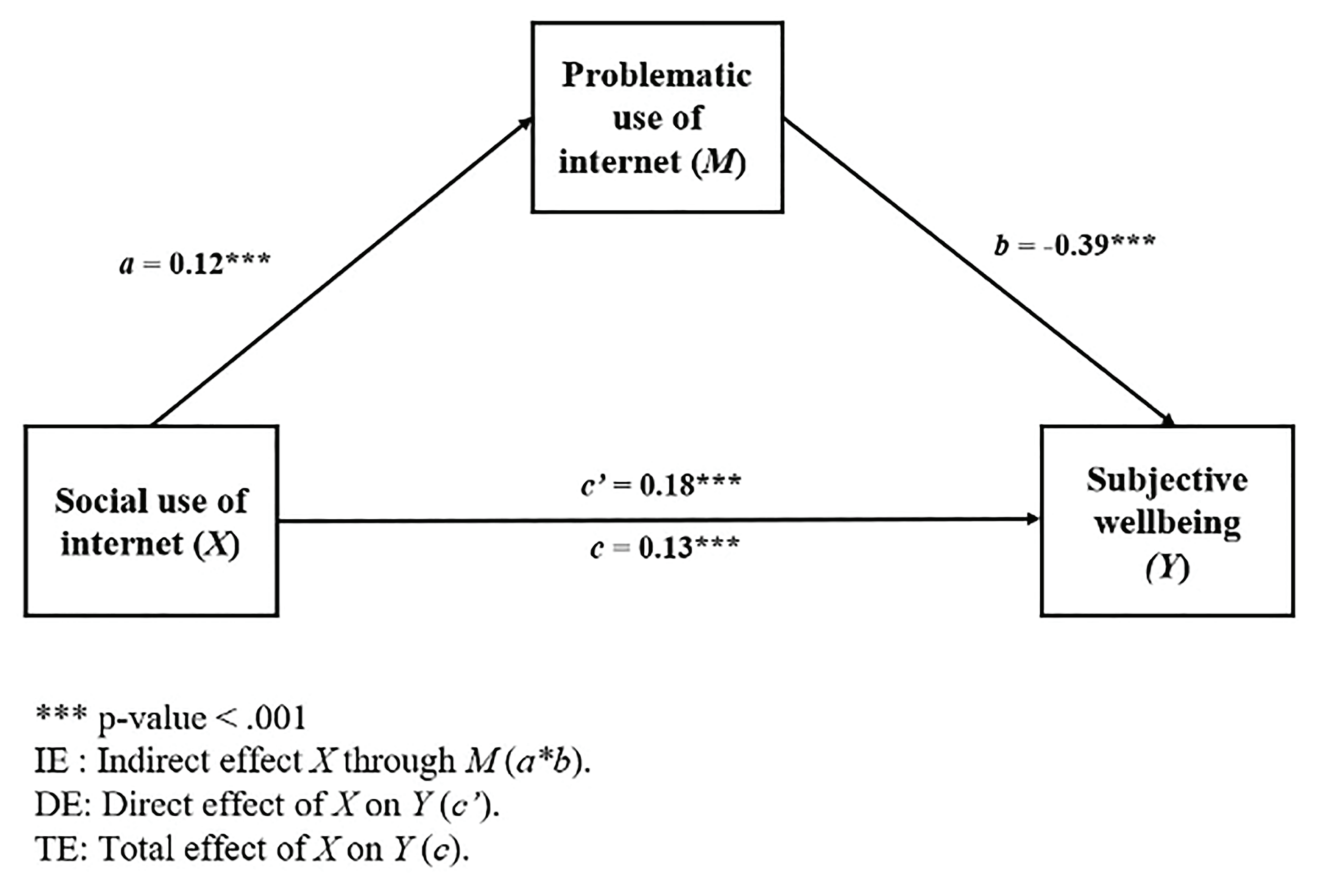
Mediational model 1.

Model 1 incorporates PUIS as a mediating variable of the relationship between social use of Internet and subjective well-being. The results of the linear regression analysis for the two sub-models that make up the mediational model are presented in Table 6, and the magnitude of the effects and directions is shown in Figure 1.

**Table 6:** Linear regression analysis for mediational Model 1.

	Consequent							
		*M* (Problematic use of Internet)				*Y* (Subjective well-being)		
Antecedent		Coeff.	*SE*	*p*		Coeff.	*SE*	*p*
*X* (Social use of Internet)	*a*	0.12	0.01	<0.001	*c'*	0.18	0.03	<0.001
*M* (Problematic use of Internet)		-	-	-	*b*	−0.39	0.04	<0.001
Sex		0.06	0.03	0.09		0.33	0.07	<0.001
Age		0.00	0.02	0.92		−0.15	0.04	<0.001
Socioeconomic status		0.08	0.06	0.20		−0.21	0.14	0.12
Cohort		0.02	0.04	0.63		0.03	0.08	0.75
Constant	*i_M_*	1.41	0.30	<0.001	*i_Y_*	10.28	0.65	<0.001
				
		*R*^2^ = 0.03				*R*^2^ = 0.05		
		*F*(5, 2,505) = 17.88, *p* < 0.001				*F*(6, 2,504) = 23.16, *p* < 0.001		

As seen in the model, the total effect of social Internet use (SUIS) on subjective well-being was statistically significant [TE: *b* = 0.13, 95% BCa CI (0.08, 0.19)], as in the cases of the individual control variables sex (*β* = 0.33, *p* < 0.001) and age (*β* = −0.15, *p* < 0.001). On the contrary, the variables SES and cohort of admission to the program were not significant.

Also, when breaking down the total effect, it is observed that both the direct effect of SUIS on subjective well-being [DE: *b* = 0.18, 95% BCa CI (0.12, 0.24)] and the indirect effect through the problematic use of the Internet were statistically significant [IE: *b* = −0.05, 95% BCa CI (−0.06, −0.03)]. A partial mediation is observed, in which the direct effect (positive) increases with respect to the total effect, while the indirect effect is negative (Figure 1). In other words, when the PUIS effect is isolated, the relationship between SUIS and PWI is still positive, but in cases that present a high problematic use, a negative effect on the students’ subjective well-being is observable.

Furthermore, Model 2 incorporated problematic use of Internet (PUIS) as the mediator of the relationship between recreational use of Internet (RUIS) and subjective well-being. Table 7 shows the results of the linear regression analysis, and their respective two sub-models, that make up the second mediational model.

**Table 7:** Linear regression analysis for mediational Model 2.

	Consequent							
		*M* (Problematic use of Internet)				*Y* (Subjective well-being)		
Antecedent		Coeff.	*SE*	*p*		Coeff.	*SE*	*p*
*X* (Recreational use of Internet)	*a*	0.11	0.30	<0.001	*c'*	0.19	0.03	<0.001
*M* (Problematic use of Internet)		-	-	-	*b*	−0.38	0.04	<0.001
Sex		0.04	0.03	0.24		0.31	0.07	<0.001
Age		0.00	0.02	0.85		−0.14	0.04	<0.01
Socioeconomic status		0.07	0.06	0.24		−0.22	0.14	0.10
Time since the student received the computer		0.01	0.02	0.86		0.02	0.08	0.84
Constant	*i_M_*	1.31	0.30	<0.001	*i_Y_*	10.04	0.66	<0.001
								
		*R*^2^ = 0.03				*R*^2^ = 0.05		
		*F*(5, 2,505) = 14.46, *p* < 0.001				*F*(6, 2,504) = 22.88, *p* < 0.001		

**Figure 2:**
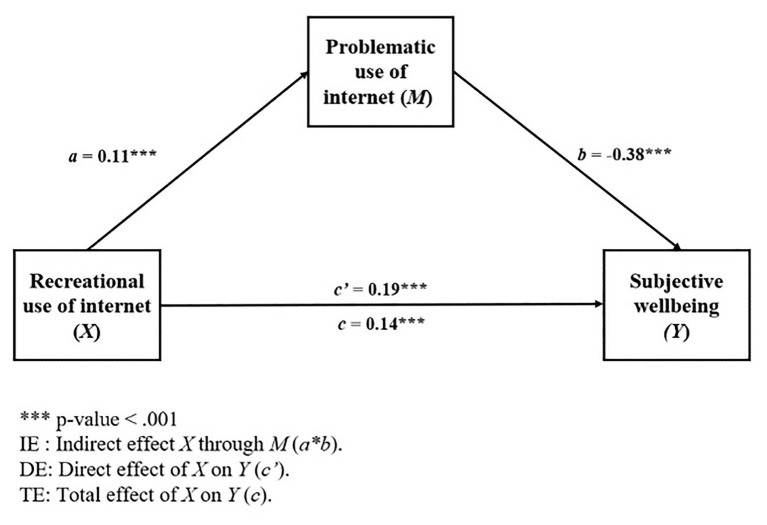
Mediational model 2.

Figure 2 presents the regression coefficients of each of the components of the different pathways presented by the model, considering the direction and magnitude of indirect, direct, and total effect over the dependent variable.

The total effect of recreational Internet use (RUIS) on subjective well-being was statistically significant [TE: *b* = 0.14, 95% BCa CI (0.08, 0.20)], and once the total effect was broken down, it was observed that both the direct effect of RUIS on subjective well-being [DE: *b* = 0.19, 95% BCa CI (0.13, 0.25)] and the indirect effect through PUIS [IE: *b* = −0.04, 95% BCa CI (−0.06, −0.03)] were statistically significant as well. The individual control variables sex (*β* = 0.31, *p* < 0.001) and age (*β* = −0.14, *p* < 0.001) were statistically significant, but the variables SES and cohort of admission to the program were not.

As in Model 1, a partial mediation was observed in Model 2, in which the direct effect (positive) increased with respect to the total effect, while the indirect effect was negative. Namely, as the recreational use of Internet increases, subjective well-being of students does in turn. However, as in Model 1, in those cases that present problematic use perceived as high, a negative effect on their subjective well-being is detected.

**Figure 3:**
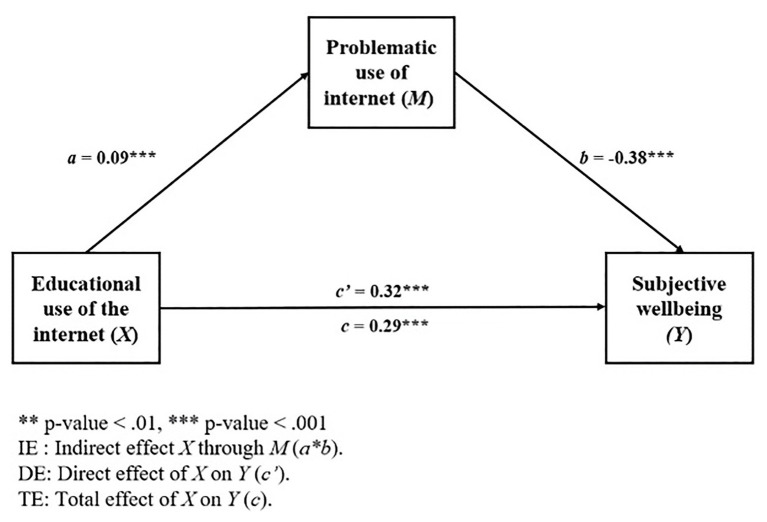
Mediational model 3.

A third model was built, placing problematic use of Internet as a mediator of the relationship between educational use of the Internet (EUIS) and subjective well-being. The results of Model 3 are presented in Table 8. The scheme of the model is shown in Figure 3.

**Table 8:** Linear regression analysis for mediational Model 3.

	Consequent							
		*M* (Problematic use of Internet)				*Y* (Subjective well-being)		
Antecedent		Coeff.	*SE*	*p*		Coeff.	*SE*	*p*
*X* (Educational use of the Internet)	*a*	0.09	0.02	<0.001	*c'*	0.29	0.04	<0.001
*M* (Problematic use of Internet)		-	-	-	*b*	−0.38	0.04	<0.001
Sex		0.06	0.03	0.08		0.35	0.07	<0.001
Age		0.00	0.02	0.98		−0.15	0.04	<0.001
Socioeconomic status		0.07	0.06	0.29		−0.24	0.13	0.08
Cohort		0.02	0.04	0.66		0.03	0.08	0.75
Constant	*i_M_*	1.51	0.30	<0.001	*i_Y_*	9.82	0.65	<0.001
								
		*R*^2^ = 0.01				*R*^2^ = 0.07		
		*F*(5, 2,505) = 6.32, *p* < 0.001				*F*(6, 2,504) = 29.15, *p* < 0.001		

As observed in the two models examined above, the total effect of educational Internet use on subjective well-being was statistically significant [TE: *b* = 0.29, 95% BCa CI (0.21, 0.36)]. Also, it was observed that both the direct effect of the educational use of the Internet on subjective well-being [DE: *b* = 0.32, 95% BCa CI (0.25, 0.40)] and the indirect effect through the problematic use of the Internet [IE: *b* = −0.03, 95% BCa CI (−0.05, −0.02)] were statistically significant when the total effect was broken down.

Regarding the control variables, the same behavior was observed as in the two previous models. That is, sex (*β* = 0.35, *p* < 0.001) and age (*β* = −0.15, *p* < 0.001) were significant, but SES and cohort of admission to the program were not.

Thereby, as in the two previous models, in Model 3, a partial mediation was observed, in which the direct effect (positive) of the independent variable (EUIS) increased for the total effect over the dependent variable (PWI), and the indirect effect (PUIS) was negative. Concretely, as the educational use of Internet increases, subjective well-being increases in turn, except in those cases where, as in the two previous models, a problematic use high enough is shown, resulting in a negative effect on the subjective well-being.

When comparing the variance explained by the mediating variable, that is, problematic use of the Internet, this reaches 3.44% in the three models built. However, when comparing the explained variance by each specific scale, the results vary according to the type of use. Indeed, for the social and recreational use scales, the relative weight in the explained variance of the model is 1.59 and 1.72%, respectively, but for educational Internet use, the explained variance rises to 2.90%.

Finally, if a replacement exercise is performed in the regression equations of each model, and two hypothetical cases are raised, where, on the one hand, a case A, corresponding to a male student, age according to sample’s mean and registering average scores across all scales of use of Internet (SUIS, RUIS, and EUIS), as well as average problem use (PUIS). On the other hand, a case B, a student with identical characteristics to case A but with a PUIS one standard deviation above the mean. Case A would obtain a PWI of 8.34, 8.38, and 8.30, respectively. Case B would obtain PWI scores of 8.02, 8.06, and 7.99, respectively. In other words, a student who presents a problematic use over the mean will register subjective well-being 3.8% (average) lower than his peers who present an average PUIS in any of the three scenarios of Internet use examined.

## Discussion

The results of the analyses allowed us to verify that the subjective well-being of students tends to decrease with age, in accordance with diverse evidence in the literature, which indicates that it decreases after 10 years (Casas and González-Carrasco, 2019; Casas et al., 2020). Also, another identifiable trend was verified in the recent discussion, and that is that female students have lower subjective well-being than male students in this same age range (Casas and González-Carrasco, 2019, 2020). When comparing the scores obtained by both male and female students, when it comes to social (SUIS) and educational (EUIS) use of the Internet, as well as problematic use (PUIS), no statistically significant differences were found. However, in the case of the recreational use (RUIS), where the difference was statistically significant, this was a minor effect. That is, when the effect of any additional variable is not controlled for, boys and girls do not present a considerably different behavior in the different types of Internet use nor in the problematic use of the Internet, a finding consistent with other studies applied to comparable populations (Cabello et al., 2020). Despite recent evidence that shows differences in intensity of use according to gender (Twenge and Martin, 2020), it was found in the present work that these are not necessarily plain to see but are more clearly manifested when the effect is mediated or controlled by more variables (Paez et al., 2020).

Regarding hypothesis No. 1 of the study, that is, that personal use of technology negatively affects the subjective well-being of schoolchildren only if its use is problematic, a relevant finding was that subjective well-being (PWI) is significantly and positively correlated with the three types of Internet use examined and negatively with the PUIS. It was also found that there is a positive and significant correlation between subjective well-being and a shorter time elapsed since the student received the equipment assigned by the MCPA program, which can be explained by the direct relationship between the cohort of entry to the program and a minor age at the time of receipt (Casas and González-Carrasco, 2019; Casas et al., 2020), as well as the effect of initial enthusiasm toward the device that may tend to diminish over time. Similarly, it was found that, as the SES rises, subjective well-being tends to increase and, at the same time, the frequency of use of technology by students tends to decrease. This may be due to the parental mediation effect that is identified in the groups with higher SES, where more spirited active and passive controls are exercised over the use of technology (Odgers, 2016; Cabello-Hutt et al., 2018).

With the aim of making a more precise distinction, the results obtained in the different tests suggest that those students who present low problematic use show higher subjective well-being than those who present a high problematic use, regardless of the grouping variable through which the contrast is carried out, whether it is sex, SES, or cohort. This difference reaches, on average, 6.8% of the PWI score.

Additionally, it was corroborated that, when schoolchildren are grouped according to the intensity of the PUIS (high and low), the general trend that female students register lower subjective well-being than their male peers is maintained, regardless of whether they belong to the low or high problematic use group. Likewise, both the RUIS and the PUIS are positively correlated with the male students. In other words, boys tend to use the Internet more intensively for recreational purposes and, at the same time, present a more problematic use than girls. Although this is consistent with the literature (García-Oliva et al., 2017), as already mentioned, it is also possible to find evidence to the contrary (Malo-Cerrato et al., 2018; Martín-Perpiñá et al., 2019). In this sense, it is possible to argue that, even when controlling for the effect of PUIS on the student population, female students register a lower PWI than male students. Likewise, this variable seems to explain better its relationship with subjective well-being than the intensity of Internet use, regardless of its purpose, and reaffirms the proposition that the sole frequency of use nor the length of time spent in front of the screen are sufficient approximations. Nevertheless, the disturbance they exert in the daily life of schoolchildren should be considered, especially in personal and social aspects (Castellana Rosell et al., 2007; Viñas Poch, 2009; Smahel et al., 2012; Rial and Gómez Salgado, 2018).

In order to fulfill the objective of the study, three multivariate models of simple mediation were also constructed, which assumed the subjective well-being of the students (PWI) as the dependent variable and the PUIS as the mediating variable. Each model incorporated a specific use scale (SUIS, RUIS, and EUIS) to its corresponding set of independent variables, which allowed a separate analysis of the effect of the type of Internet use on subjective well-being. Although the variance of the dependent variable explained by independent ones is apparently low (5–7% for the three models), it must be pointed out that subjective well-being is a complex variable, and it is affected by almost all the variables that surround the context of the individuals. For this reason, it should be considered, since it shows that the model is successful in identifying the effect that different types of Internet use have on subjective well-being, especially considering that all the models were statistically significant.

In the three models constructed, the simple mediation analysis results indicated that only if the intensity of Internet use is expressed as problematic does it have a negative effect on subjective well-being. On the contrary, when Internet use is not associated with problematic use, the effect is positive and even greater than the simple effect (without mediation) between these two variables. In other words, high intensity of use does not mean that it is necessarily problematic, but rather that they are phenomena that act independently. In simple words, when the PUIS effect is isolated, the relationship between the different types of use of Internet and PWI is positive, namely, as the use of the Internet increases, subjective well-being increases. Nevertheless, in those cases that present problematic use high enough, this makes a negative effect on the subjective well-being. This is one of the main findings of the present research.

Regarding hypothesis 2, specifically, the effect on subjective well-being is different according to the type of Internet use, it was possible to corroborate that, by isolating the magnitude of the effect of problematic use, the different types of use studied make a significant contribution to the variance explained by the respective model, but their magnitudes are different. Thus, social and recreational use represent 1.59 and 1.72% of the variance explained in their respective models, but, in the case of educational use, the variance explained by this rises to 3.44%.

Therefore, the evidence collected allows us to sustain that the intense use of the Internet, whether for social, recreational, and, especially, educational purposes, as long as it is not problematic, has a positive effect on students’ subjective well-being. The latter complements previous research that detected that, by separating the intensity of Internet use for educational purposes from those for general purposes, the relationship with academic performance is positive (Kim et al., 2017). In addition, it allows to reaffirm the proposition that technology is not neutral (Zhao et al., 2004; Delvenne and Parotte, 2019) and that its different types of use have, in turn, different impacts on the lives of people.

Until now, the major limitations of recent research are that it concentrates mainly on Internet access of schoolchildren in developed countries and that it focuses mainly on the social application they give to it (Odgers, 2016; Beyens et al., 2020; George et al., 2020; Luo and Hancock, 2020; Mylonopoulos and Theoharakis, 2020; Walsh et al., 2020). Meanwhile, it seems to neglect Internet recreational use (Przybylski and Weinstein, 2017), and it virtually ignores the influence that the educational use of it could have on the well-being of students. In this sense, the relevance of the findings provided by this study lies in the fact that they allow for discussion that, in order to investigate in depth the effect that the intensity of Internet use can have on the subjective well-being of schoolchildren, first, the mediating effect exerted by the problematic use of the Internet must be taken into account, second, that not all types of use have the same impact on subjective well-being, and, third, that since digital development is not distributed equitably in the world (Hilbert, 2016; Third et al., 2017), it is crucial to critically evaluate the approaches that omit the existence of the digital divide when investigating the possible consequences of Internet use.

Although there is consensus in the specialized discussion (Ivie et al., 2020; Kardefelt-Winther et al., 2020; Young et al., 2020) that the different apparently contradictory results reflect that the phenomenon has not yet been sufficiently studied (Przybylski and Weinstein, 2017; Orben and Przybylski, 2019; Young et al., 2020), it is necessary to consider that there are sociocultural, technological, and economic differences at country level that are not being taken into account (Diener et al., 2018) in the study of the relationship between digital technologies and subjective well-being. This first-world bias could be one of the multiple causes that explain the difficulty of obtaining conclusions that can be generalized to broader realities, especially to less economically and digitally developed regions (Laugasson et al., 2016; Donoso et al., 2021).

One of the main limitations of this study is that, given that the cases come from the beneficiaries of a focused public policy, the sample shows a slight prevalence of students belonging to the lowest SESs, which may not be adjusted to what happens in high-income groups. Contexts with high segregated school systems like the one of Chile (González and Fernández-Vergara, 2019; Bellei et al., 2020), where private schools have only students with high SES, and public schools the opposite, are to be studied in further research. Another limitation is that the use of self-report in schoolchildren should always be taken with caution, as it might limit the accuracy of the data. It is to be noted that the type and frequency of Internet use were obtained through this modality for this research. Moreover, this work did not consider dependence, compulsiveness, or any other psychological problems that may be related to the study variables. Finally, it is worth mentioning, due to the cross-sectional design of the study, that these results should not be considered as a causal relationship.

Finally, considering that the first- and second-generation digital divide continues to be substantial in developing countries and that programs for the direct delivery of technological equipment to schoolchildren continue to exist in these regions (UNICEF, 2017; Cabello et al., 2018), it is important that future research monitors the possible effects that these projects may have not only on subjective well-being, the sense of belonging and social integration of schoolchildren, but also on the rest of the family group and the educational community, given that, in many cases, access to these devices and their connectivity possibilities constitute the first approach to the digital world, especially in the lower-income population. Therefore, it is essential to highlight the role that technology can have in education not only in its potential contribution to obtaining better learning results but also in its contribution to the opening of various opportunities for growth, integration, and belonging to which children and adolescents should have access to as part of their development process. Last but not least, given that this type of initiative represents a very significant expense, both from public budget as well as donations from the technology industry, we suggest observing the results from a broader perspective with the aim of diversifying the approach used to evaluate the social impact of these efforts and to deepen scientific research on the matter.

In conclusion, although the discussion about the problematic use of technology, particularly the Internet, is still in progress, the results of this study provide inputs that allow advancing toward a more comprehensive understanding of the differentiated effects than the type or purpose of use have on the subjective well-being of adolescents. Likewise, the findings of this research allow us to affirm that intense use, but which does not qualify as problematic, not only does not have a negative effect on subjective well-being, but on the contrary, has a positive one. In other words, distinguishing the purposes of use of digital technologies is vital to draw more precise conclusions in the study of subjective well-being.

## Data Availability Statement

The data analyzed in this study is subject to the following licenses/restrictions: Dataset is only available upon request, due to the policy of the Ministry of Education of Chile. Requests to access these datasets should be directed to Centro de Estudios Mineduc, estudios@mineduc.cl.

## Ethics Statement

The studies involving human participants were reviewed and approved by Enlaces, Education and Technology Center, Ministry of Education of Chile. Written informed consent to participate in this study was provided by the participants’ legal guardian/next of kin.

## Author Contributions

GD contributed to the investigation, data curation, formal analysis, and writing the original draft. FC contributed to the conceptualization, formal analysis, writing, review, and editing. AR contributed to the methodology, formal analysis, writing, review, and editing. CC contributed to the writing, review, editing, and translation. All authors contributed to the article and approved the submitted version.

### Conflict of Interest

The authors declare that the research was conducted in the absence of any commercial or financial relationships that could be construed as a potential conflict of interest.

The handling Editor declared a past co-authorship with one of the authors AR.
